# The sound of soft alcohol: Crossmodal associations between interjections and liquor

**DOI:** 10.1371/journal.pone.0220449

**Published:** 2019-08-08

**Authors:** Bodo Winter, Paula Pérez-Sobrino, Lucien Brown

**Affiliations:** 1 Department of English Language & Linguistics, University of Birmingham, Birmingham, United Kingdom; 2 Applied Linguistics Department, Universidad Politécnica de Madrid, Madrid, Spain; 3 Korean Studies Program, Monash University, Melbourne, Australia; Northumbria University, UNITED KINGDOM

## Abstract

An increasing number of studies reveal crossmodal correspondences between speech sounds and perceptual features such as shape and size. In this study, we show that an interjection Koreans produce when downing a shot of liquor reliably triggers crossmodal associations in American English, German, Spanish, and Chinese listeners who do not speak Korean. Based on how this sound is used in advertising campaigns for the Korean liquor soju, we derive predictions for different crossmodal associations. Our experiments show that the same speech sound is reliably associated with various perceptual, affective, and social meanings. This demonstrates what we call the ‘pluripotentiality’ of iconicity, that is, the same speech sound is able to trigger a web of interrelated mental associations across different dimensions. We argue that the specific semantic associations evoked by iconic stimuli depend on the task, with iconic meanings having a ‘latent’ quality that becomes ‘actual’ in specific semantic contexts. We outline implications for theories of iconicity and advertising.

## Introduction

Iconicity refers to the resemblance between the form of a linguistic signal and its meaning. For example, onomatopoetic words such as *screech*, *beep* and *bang* resemble the sounds they describe. Iconicity also involves crossmodal associations, such as when the high front vowel /i/ is associated with the perceptual idea of “smallness” [1–3]. Similarly, the made-up words *kiki* and *takete* are reliably matched to spiky shapes, whereas *bouba* and *maluma* are matched to round shapes [4–8]. In stark contrast to the traditional assumption that languages are dominated by the principle of arbitrariness (no direct connection between form and meaning), more and more research is uncovering that languages harbor a considerable degree of iconicity in their vocabularies [1,9–12].

Researchers have already noted that iconicity has applications for the development of product and brand names [13–17]. For example, participants associate the hypothetical name *Nullen* with a thicker type of ketchup than *Nellen*, they associate *Usab* with a darker beer than *Esab* [13], and they associate *Frosh* with a creamier ice cream than *Frish* [18]. These studies clearly show that consumers use the name of a product to make inferences about its qualities, which is relevant to product valuation as we know that consumers generally like to see their expectations confirmed [19].

In most studies on iconicity in advertising, researchers exploit iconicity in brand names by creating artificial words to see how these are associated with specific product dimensions. Here, we do the reverse: we take a phonological contrast that is attested in an actual advertising campaign, using the advertising campaign to derive predictions for different experiments. Rather than associations between speech sounds and single perceptual dimensions, we are specifically interested in the capacity of iconicity to involve multiple crossmodal associations. Research in the field of metaphor in advertising can illustrate this point. Experts have proposed that one of the advantages conferred by metaphors in advertising is that they allow [20] “multiple, distinct, positive inferences about the advertised brand” (p. 7). That is, metaphors can highlight a product’s qualities in an indirect fashion, with one and the same metaphor having multiple loose interpretations that the consumer is able to select from and/or enrich for themselves [20–22]. We want to suggest that iconicity might also work like this. For example, the pseudoword *kiki* (as opposed to *bouba*) does not inherently mean “spikey” unless the task highlights this dimension. In other contexts, the high front vowel /i/ is associated with smallness or bitter taste [23,24]. Thus, we envision iconicity as *latent* and *pluripotential*—the same phonological pattern can have multiple distinct meanings, depending on the context.

In this study, we explore this latent and pluripotential quality of iconicity by using an ecologically motivated example from a Korean advertising campaign. Harkness [25] details the multiple semiotic strategies Korean companies use to advertise the “softness” of Korea’s most famous liquor, soju. This liquor takes up 97% of the spirits market in South Korea and is frequently claimed to be one of the most popular spirits of the world (The Guardian, https://www.theguardian.com/lifeandstyle/wordofmouth/2013/dec/02/soju-popular-booze-world-south-korea, accessed July 27, 2019). What is relevant for the present discussion is that soju has undergone drastic changes over recent decades. Whereas most sojus in the 1980s were 30–35% in alcohol content, 20% is now considered standard strength, with some fruit-flavored sojus going as far down as 14%.

Harkness [25] details how alongside this deliberate diluting of soju to gain mass appeal, particularly from female customers, there were a number of changes in advertising strategy. For example, a particular 1982 commercial for soju of the Bohae brand showed middle-aged men on a construction site eating skewers of meat against the backdrop of a bonfire. A male voice-over with a deep voice advertised the soju with heavy guitar music playing in the background; the entire color palette of the commercial was soaked in brown and red. Compared to this, modern soju commercials emphasizing its “soft” qualities often feature energetic young women, frequently speaking with stylized, high-pitched voices. The color palettes have changed to light green and blue, signaling freshness and building up associations with spring and nature.

Amidst these changes, the Jinro company also used a form of what they themselves called “voice marketing” during a 2008 advertising campaign for their Chamisul brand of soju [25,26]. In Korean culture, after somebody downs a shot of soju, they may utter an interjection that in English can be written as *khu* (in Korean script: 크; in the International Phonetic Alphabet: [k^h^ɯ]). This sound is produced with an aspirated velar stop (a consonant produced at the back of the mouth) and a high back vowel that is unrounded (produced at the same position as [u], but without lip rounding). Koreans may utter this sound as a vocal gesture of disgust, or in this case to index the bitterness or intensity of the alcohol they just tasted. However, the Chamisul brand deliberately avoided *khu* in their “voice marketing” and instead used the alternative variant *khya* (캬, [k^h^ja]) on its print ads. *Khya* is a recent innovation which Harkness [18] describes as representing a “softening and brightening” of *khu* (p. 20). This variant form indexes a milder taste, a lighter, less intense drinking experience and, according to Harkness [18], is preferred by female drinkers and increasingly by younger men too (p. 21). In contrast to *khu*, the “softer” variant *khya* is produced with an open low-back vowel [a]. Other soju brands have also made use of these sounds in their advertising, including a 2015 television commercial for the citrus-flavored soju Sunhari Chum Churum produced by Lotte Chilsung Beverage.

If the *khya/khu* contrast is indeed iconic, this would fit with various studies that have found reliable associations between speech sounds and tastes [27,28]. For example, Gallace and colleagues [29] found that potato chips are judged to be more *takete* than brie cheese, whereas *maluma* is reliably associated with sweet tastes [30].

In our experiments, we detach the *khya/khu* sounds from their local Korean context and play them to naïve American English, German, Spanish and Chinese listeners who have relatively little to no exposure to Korean culture. We want to know whether these two sounds have multisensory associations consistent with the “voice marketing” campaign of the Jinro company, and whether these associations are accessible to non-Korean speakers. If listeners from other cultures have the same mental associations as evidenced by Korean marketing campaigns, this would suggest that the *khya/khu* contrast is indeed iconic, since these listeners have no arbitrary language-specific conventions to base their judgments on. Our experiments thus follow the frequently adopted strategy to demonstrate iconicity via testing the cross-cultural transparency of particular sound patterns [31–35]. In doing so, our study enriches understanding of iconicity as a marketing strategy since the *khya/khu* contrast is an ecologically valid example of a sound distinction being actively used in real-world marketing for major liquor brands. Previous experimental research on iconicity in marketing has tended to use only made-up words, and it has tended to focus only on brand names [13,18]. In addition, we demonstrate *latent iconicity* and *pluripotentiality* by showing that the same contrast (*khya* versus *khu*) has multiple distinct sensory, emotional, and social associations.

For the following experiments, we asked two Korean native speakers (one male, one female) two produce the *khya*/*khu* interjections while simultaneously imagining that they had just downed a shot of soju. These two sounds were then used for a series of experiments where we vary the semantic dimension tested, with predictions for the different dimensions derived from the Korean advertising campaigns.

Specifically, we asked American English native speakers which ones of the two sounds is “softer” or “harder” (Experiment 1), based on Harkness’s description of *khya* being a “softening” of *khu* [25]. In Experiment 2, we extended this task from hardness/softness to roughness/smoothness, which is the most salient dimension of touch [36,37] that is furthermore perceptually associated with the “hardness”/”softness” dimension [38–42]. In Experiment 3, we extended the task to the social dimension of gender, asking participants which one of the two sounds is more “male” or “female”. Here, following Harkness’s account of the Korean advertising campaigns [25], we expect *khya* to be perceived as more female than *khu*, which correspondingly should be more strongly associated with masculinity.

For Experiment 4, we tested the dimension of alcohol content, asking participants which one of the two sounds fits better with a 15% or 30% liquor. Following the use of these sounds in Korean advertising campaigns, we expect *khu* to be more strongly associated with the 30% liquor than *khya*, which should be more strongly associated with the weaker liquor. Experiment 5 extends the task to the domain of taste by asking participants which on the two sounds is more “bitter” or “sweet” [30].

Finally, Experiment 6 extends the task to the dimension of pleasantness, where we predict *khya* to be more pleasant than *khu*. This prediction is motivated because several of the other dimensions tested in the other experiments have pleasantness (or unpleasantness) as a common denominator. For example, studies found that people find soft and smooth surfaces more pleasant than hard and rough ones [43–47], and sweet tastes are preferred over bitter tastes [48–50]. More generally, Experiment 6 is motivated by the fact that there is an increasing number of studies finding evidence for the presence of affective iconicity in language, i.e., the association between speech sounds and pleasant or unpleasant feelings [51–55].

After demonstrating latent iconicity and pluripotentiality in this cross-linguistic listening task for American English listeners, we then replicate the association between *khya*/*khu* with softness/hardness tested in Experiment 1 in a listening experiment with Spanish, German, and Chinese listeners.

## Experiments: General methods

This study was approved by the University of Birmingham Research Humanities and Social Sciences Ethical Review Committee (ERN_17–0040). Consent was obtained electronically.

### Participants

For the first series of experiments, all of our participants were native speakers of American English. Table 1 lists the participants across the different studies and their ages.

**Table 1:** Breakdown of participants.

	Participants	Female	Male	Mean age	Age range
**Experiment 1**	110	51	59	34	19–69
**Experiment 2**	108	47	61	34	19–66
**Experiment 3**	104	48	56	35	19–66
**Experiment 4**	104	52	52	33	19–71
**Experiment 5**	201	89	112	34	19–68
**Experiment 6**	201	83	118	34	18–70
**Total**	828	370	458		

All participants were recruited via Amazon Mechanical Turk and received 0.25 USD reimbursement. Amazon Mechanical Turk is known to be a valid tool for collecting behavioral data [56–58] and performing linguistic experiments [59]. Approval rate is a strong factor in data quality [60], and so we only allowed workers with an approval rate of 93% or above to participate in our study.

Since we are interested in the responses of naïve listeners, we excluded participants that had spent time in Korea and/or that had studied Korean as a second language. We had to exclude 10 participants (9%) for Experiment 1, 11 participants (10%) for Experiment 2, 7 participants (7%) for Experiment 3, 8 participants (8%) for Experiment 4, 48 participants (24%) for Experiment 5, and 65 (32%) for Experiment 6 because of these criteria. We additionally assessed whether the results held with even stricter exclusion criteria, where we excluded all participants who indicated to have tried soju or who reported to have at least one Korean friend. All results below hold even if these stricter exclusion criteria are applied.

### Stimuli

The *khu* and *khya* utterances were recorded with Praat (created by Paul Boersma and David Weenink, http://www.fon.hum.uva.nl/praat/) on a MacBook Air using a head-mounted microphone. Both speakers (one male, one female) were 25 years old and self-reported to be speakers of the Seoul dialect. For each speaker, the two stimuli were approximately equal in length (female *khu* 672ms, *khya* 697ms; male: 687 / 691ms) and amplitude (female: 78dB / 72dB, male: 75dB / 77 dB). For the listening experiment, we added a 300ms silent pause to the beginning of each stimulus.

It should be emphasized that the *khu* and *khya* renditions we got from our speakers were, in fact, entirely voiceless. That is, there was no glottal fold vibration and no pitch associated with them. In addition, there was no palatalization of the *khya* sound, i.e., the sound more resembled the transliteration *kha*. Due to the absence of any voicing, participants must make any judgments on these sounds based on the spectral characteristics of the sounds, not pitch.

### Experimental procedure

All experiments were run online via Qualtrics. Participants were instructed as follows:

“On the next screen, you will be presented with two audio files. Listen to both files and answer a question. We prefer that you listen to each file only once, but you are free to listen multiple times.”

After clicking NEXT, one of two trials appeared on the next screen with two sound files that had to be clicked in order to be played. The sound files were labeled “first” and “second.” Below the sound files, we presented the question, “Which one sounds ‘softer’, the first or the second?”, on top of two buttons labelled “First” and “Second”. In Experiment 1, for half of the participants, the question was changed so that participants were asked which sound file sounded “harder” rather than “softer”. Thus, the main condition variable (soft versus hard) was a between-participants variable. Each person was played the male and female pairs back-to-back, with male and female versions presented in a randomized order. Within each pair, the order of *khya* and *khu* was also randomized.

The basic setup was used for all other experiments, except for Experiment 4 on alcohol content. In contrast to dichotomies such as “hard” and “soft,” alcohol content has no associated verbal antonyms. We thus asked the question “Which sound do you associate with the 15% liquor?” to half of the participants, and the question “Which sound do you associate with the 30% liquor?” to the other half. However, to make sure that there is some form of binary opposition (consistent with the task), we told participants that they need to compare two liquors: one that was 15% and one that was 30%.

The precise percentage values chosen for the alcohol study approximate the shift in the alcohol content of soju from the typical 1980s versions to the low alcohol sojus of today. We also felt the need to contextualize the task to participants with more detail as alcohol content frequently correlates with taste properties (for example, beverages with around 5% are often beers and beverages with around 12% are often wines). To reduce the impact of these secondary associations, we told participants that the 15% and 30% liquor were identical in taste and only differed in alcohol content. In addition, we referred to the drinks as “rice liquor” because we presumed that it would be unfamiliar to our English-speaking participants, who would therefore not have any strong preconception about taste. It should be easier for participants to imagine that unfamiliar liquors at 15% and 30% are similar in taste, compared to, for example, 15% and 30% wines.

The reason for conducting a between-participants experiment, and furthermore for splitting the different semantic dimensions across different experiments, was that we wanted responses to be as uninfluenced from each other than possible. If a participant, for example, chose the “soft” option for *khya* on a first trial, and then was asked which one was “smooth,” their response would likely be influenced by the previous choice. Moreover, the task presumably felt quite abstract to participants (who have never heard these sounds and probably thought that they are guessing), which is why we wanted to make the experiment as short as possible. Thus, to avoid order effects and to keep the experiment short, all factors in our experiment (except for the Voice Gender variable) were between-participants factors.

### Statistical analyses

For all statistical analyses, we used the R statistical programming environment version 3.3.1. All data and code are available in the following Open Science Framework repository: https://osf.io/jzqg9

Throughout all analyses reported below, we used logistic regression because our dependent measure was categorical. We furthermore used *mixed* logistic regression because our experiment included repeated measures—two responses per participant (one for each stimulus pair). We included the factor “Condition” (soft versus hard, rough versus smooth, male versus female etc.) and “Listener Gender” (male participant versus female participant) as between-participants fixed effects. We included the factor “Voice Gender” (whether the stimulus set was male or female) as within-participants fixed effect. We additionally fitted Condition * Listener Gender and Condition * Voice Gender interactions. Given the role of gender highlighted by Harkness [25], it is plausible that participants might have different preferences for *khu*/*khya* depending on whether they are male or female, and depending on whether they are listening to a male or female stimulus voice. All categorical fixed effects were sum-coded to facilitate the interpretation of interactions. We included “Listener” as random effect (no random slopes were fitted as the critical effect in question “Condition” was a between-participants variable). All *p*-values are based on likelihood ratio tests (deviance tests) of the model with the effect in question against a null model without the effect in question. Throughout the paper, we report marginal *R*^*2*^ values for the model (variance attributed to the fixed effects only).

## Results

### Experiment 1: *Softness*

**Fig 1:**
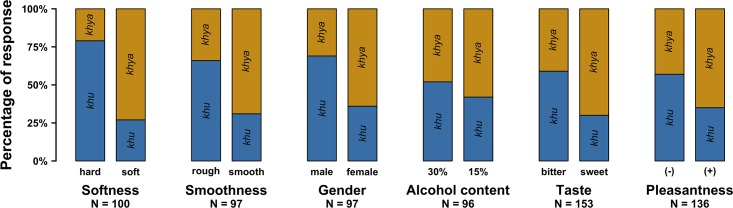
Percentage of *khu* or *khya* responses (pooled over both trials) for the softness (Experiment 1), smoothness (Experiment 2), gender (Experiment 3), alcohol content (Experiment 4), taste (Experiment 5), and pleasantness (Experiment 6) questions.

Participants who were asked “which one sounds softer?”, picked *khya* 73% of the time and *khu* 27% of the time. Participants who were asked the “harder” question showed the reverse pattern: they picked *khu* 79% of the time and *khya* 21% of the time. There was a statistically reliable main effect of Condition (*χ*^*2*^(1) = 58.21, *p* < 0.0001). Fig 1 (left two bars) displays the average proportion of *khya* and *khu* responses in relation to hard and soft questions (averaged over the male and female voices).

There were no reliable main effects of Listener Gender (*χ*^*2*^(1) = 1.46, *p* = 0.23) or Voice Gender (*χ*^*2*^(1) = 1.04, *p* = 0.31). However, there were reliable interactions between Condition and Listener Gender (*χ*^*2*^(1) = 8.2, *p* = 0.004) and Condition and Voice Gender (*χ*^*2*^(1) = 31.5, *p* < 0.0001). Specifically, male listeners showed a stronger difference between the two types of questions. Whereas men picked *khu* 85% of the time when asked the “harder” question, women picked *khu* only 64% of the time. For the voices, the gender effect went in the opposite direction: Averaged across both listener genders, participants picked *khu* 93% of the time when asked the “harder” question for the female voice stimuli but only 58% of the time for male voice stimuli. Overall, the logistic mixed effects regression model described 55% of the variance in *khu* versus *khya* choices (marginal *R*^*2*^). 54% of the overall variance in *khu*/*khya* responses could be attributed to the effect of Condition and its interactions.

The results confirm the central prediction that *khya* is associated with softness, and *khu* with hardness. This was the case even though our listeners had not been exposed to the Korean language or Korean soju-related rituals.

### Experiment 2: *Smoothness*

Participants who were asked, “which one sounds smoother?”, picked *khya* 69% of the time and *khu* 31% of the time. On the other hand, participants who were asked the “rougher” question showed the reverse pattern, picking *khu* 66% of the time and *khya* 34% of the time (see Fig 1). There was a statistically reliable main effect of Condition (*χ*^*2*^(1) = 26.02, *p* < 0.0001), as well as a reliable interaction between Condition and Voice Gender (*χ*^*2*^(1) = 10.56, *p* = 0.001). Specifically, there was again a stronger Condition effect for the female voice stimuli than for the male voice stimuli: For the female voice stimuli, 75% of all participants picked *khu* when asked the “rougher?” question. For the male voice stimuli, only 59% of all participants picked *khu* in the rough condition. This time there was no Condition and Listener Gender interaction (*χ*^*2*^(1) = 0.53, *p* = 0.47), however, there was a reliable main effect of Listener Gender (*χ*^*2*^(1) = 6.85, *p* = 0.009), with female listeners overall picking more *khya* responses (76%). There was no main effect of Voice Gender (*χ*^*2*^(1) = 1.74, *p* = 0.19). Overall, the logistic mixed effects regression model described 27% of the variance in *khu* versus *khya* choices, of which 22% could be attributed to Condition and its interactions.

Experiment 2 thus shows that the *khya/khu* contrast extends from softness/hardness to smoothness/roughness.

### Experiment 3: *Gender*

The next experiment assessed whether the present findings carry over to the social domain of gender, which would show that the *khya/khu* contrast truly has a lot of latent meaning potential; it can not only trigger sensory meaning, but also more abstract social meanings, in line with the gendered cultural meanings of soju discussed in Harkness [25].

Participants who were asked which one sounds “more female?”, picked *khya* 65% of the time and *khu* 35% of the time. On the other hand, participants who were asked the “more male” question showed the reverse pattern: they picked *khu* 70% of the time and *khya* 30% of the time (see Fig 1). There was a statistically reliable main effect of Condition (*χ*^*2*^(1) = 20.52, *p* < 0.0001). This time there were no reliable interaction effects of Condition and Voice Gender (*χ*^*2*^(1) = 1.16, *p* = 0.28) or Condition and Listener Gender (*χ*^*2*^(1) = 1.86, *p* = 0.17). There were no reliable main effects of Listener Gender (*χ*^*2*^(1) = 0.28, *p* = 0.60) and Voice Gender (*χ*^*2*^(1) = 0.59, *p* = 0.44). Overall, the logistic mixed effects regression model described 15% of the variance in *khu* versus *khya* choices, which was almost entirely due to Condition and its interactions (14.6%).

Experiment 3 showed that the *khu/khya* split that we showed for softness/hardness in Experiment 1 and smoothness/roughness in Experiment 2 successfully carried over to a question about gender. American English listeners associated *khya* more strongly with femininity than *khu*. This result is obtained with both the male and the female stimulus voices. That is, even for a male voice *khya* is perceived as more feminine, and conversely, even for a female voice *khu* is perceived as more masculine.

### Experiment 4: *Alcohol content*

In Experiment 4, we extend the pattern observed in Experiments 1–3 to the domain of alcohol content. 58% of all participants chose *khya* as opposed to *khu* when asked to match a sound with a 15% liquor. 52% of all participants chose *khu* as opposed to *khya* when asked which sound matched a 30% liquor. In this case, we failed to obtain a main effect of Condition (*χ*^*2*^(1) = 1.82, *p* = 0.18). Instead, the Condition effect content only emerged in the form of an interaction with Voice Gender (*χ*^*2*^(1) = 10.86, *p* < 0.001): the *khya/khu* split was stronger for the female stimulus pair.

There were no Voice Gender (*χ*^*2*^(1) = 1.88, *p* = 0.17) or Listener Gender (*χ*^*2*^(1) = 0.05, *p* = 0.83) main effects, as well as no Listener Gender * Condition interaction (*χ*^*2*^(1) = 0.12, *p* = 0.73). Overall, the logistic mixed effects regression model described 8.9% of the variance in responses, which is almost entirely due to the Condition effect and its interactions (7.9%).

Experiment 4 confirms our prediction only partially. While Condition did play a role, it was not in terms of being a main effect. The pattern was qualitatively similar to what has been observed in Experiments 1–3, with a stronger effect for the female voice pair than for the male voice pair.

### Experiment 5: *Taste*

Experiment 5 tests the association of *khya* and *khu* with the taste dimension of “sweet” and “bitter.” 70% of all participants chose *khya* as opposed to *khu* when asked the question which one sounds “sweeter?”, with only 30% of all participants choosing *khu* in this case. In contrast, only 41% of all participants chose *khya* when asked which one sounds “bitter?”, which conversely showed a higher percentage of *khu* responses (59%). There was a statistically reliable effect of Condition (*χ*^*2*^(1) = 24.94, *p* < 0.0001).

There were was no reliable effect of Voice Gender (*χ*^*2*^(1) = 1.54, *p* = 0.21) and Listener Gender (*χ*^*2*^(1) = 0.98, *p* = 0.32), as well as no Voice Gender * Condition (*χ*^*2*^(1) = 0.04, *p* = 0.83) or Listener Gender * Condition (*χ*^*2*^(1) = 0.63, *p* = 0.43) interactions. Overall, the logistic mixed effects regression described 12.0% of the variance in responses, which was mostly due to the Condition effect and its interactions (10.8%).

Experiment 5 thus shows that the *khya/khu* contrast extends to the dimension of taste, particularly, the opposition of “sweet” and “bitter.”

### Experiment 6: *Pleasantness*

Experiment 6 tests the extent to which the associations reported so far extend to the pleasantness/unpleasantness dimension. When asked which sound is more “pleasant”, 65% of all participants chose *khya*, as opposed to 35% who chose *khu*. Those participants who were asked which one sounds more “unpleasant”, picked *khya* 43% of the time, and *khu* 57% of the time. There was a statistically reliable effect of Condition (*χ*^*2*^(1) = 12.70, *p* = 0.0004), as well as a reliable Condition and Voice Gender interaction (*χ*^*2*^(1) = 14.22, *p* = 0.0002). As before, the *khya*/*khu* split was stronger for the female stimulus than the male stimulus.

There were no Voice Gender (*χ*^*2*^(1) = 0.24, *p* = 0.62) or Listener Gender (*χ*^*2*^(1) = 0.38, *p* = 0.54) main effects, as well as no Listener Gender * Condition interaction (*χ*^*2*^(1) = 0.85, *p* = 0.36). Overall, the logistic mixed effects regression described 12.6% of the variance in responses, which was mostly due to the Condition effect and its interactions (12.3%).

Experiment 6 thus shows that the *khya/khu* contrast extends to the dimension of pleasantness/unpleasantness.

### Experiment 7: Replication of Experiment 1 with Spanish, German, and Chinese

#### Methods

So far, Experiments 1 to 6 were conducted with a sample of native-speaking American English listeners that were recruited via Amazon Mechanical Turk. This set-up already demonstrates at least some degree of cross-linguistic and cross-cultural generality to the extent that Korean and American English are two unrelated languages (Korean is a language isolate, American English an Indo-European language) that are spoken in geographically separated areas that have distinctly different cultures. In this section, we extend the present investigation to three more languages, Spanish (a Romance language of the Indo-European family), German (another Germanic language of the Indo-European family), and Chinese (a Sino-Tibetan language). The first author (a native speaker of German) translated the English instructions of Experiment 1to German; the second author (a native speaker of Spanish) translated the the instructions to Spanish. In addition, we consulted a native speaker of Mandarin Chinese to translate the instructions to Chinese (Wangmeng Jiang). The English words *hard* and *soft* were translated into Spanish *duro* and *blando*, German *hart* and *weich*, and Chinese 更强硬 *geng qiangying* and 更柔和 *geng rouhe*.

We used a snowballing technique to recruit volunteer participants via social media. Also, additional Spanish and German listeners were recruited via Amazon Mechanical Turk and paid 0.25 USD, as in the other experiments. We ended up with a total of 167 Spanish (mean age: 35, range: 18–73), 118 German (mean age: 34; range: 18–66) and 69 Chinese speakers (mean age: 26; range: 18–36). There was a difference in exposure to Korean culture between the samples: 32% of the Chinese sample have either been to Korea at least once or learning Korean; for the German sample, this figure was 20%; for the Spanish sample this was 7%. As excluding these speakers results in a very small sample size, particularly for the Chinese sample, the results below are reported for the entire sample (no exclusion). However, the main condition effect (hard versus soft) is statistically reliable (*p* < 0.05) even if everybody who has been to Korea or has experience learning Korean is excluded from the samples.

We fitted logistic mixed effects regression models of exactly the same structure as with the American English *hard*/*soft* study.

#### Results

**Fig 2:**
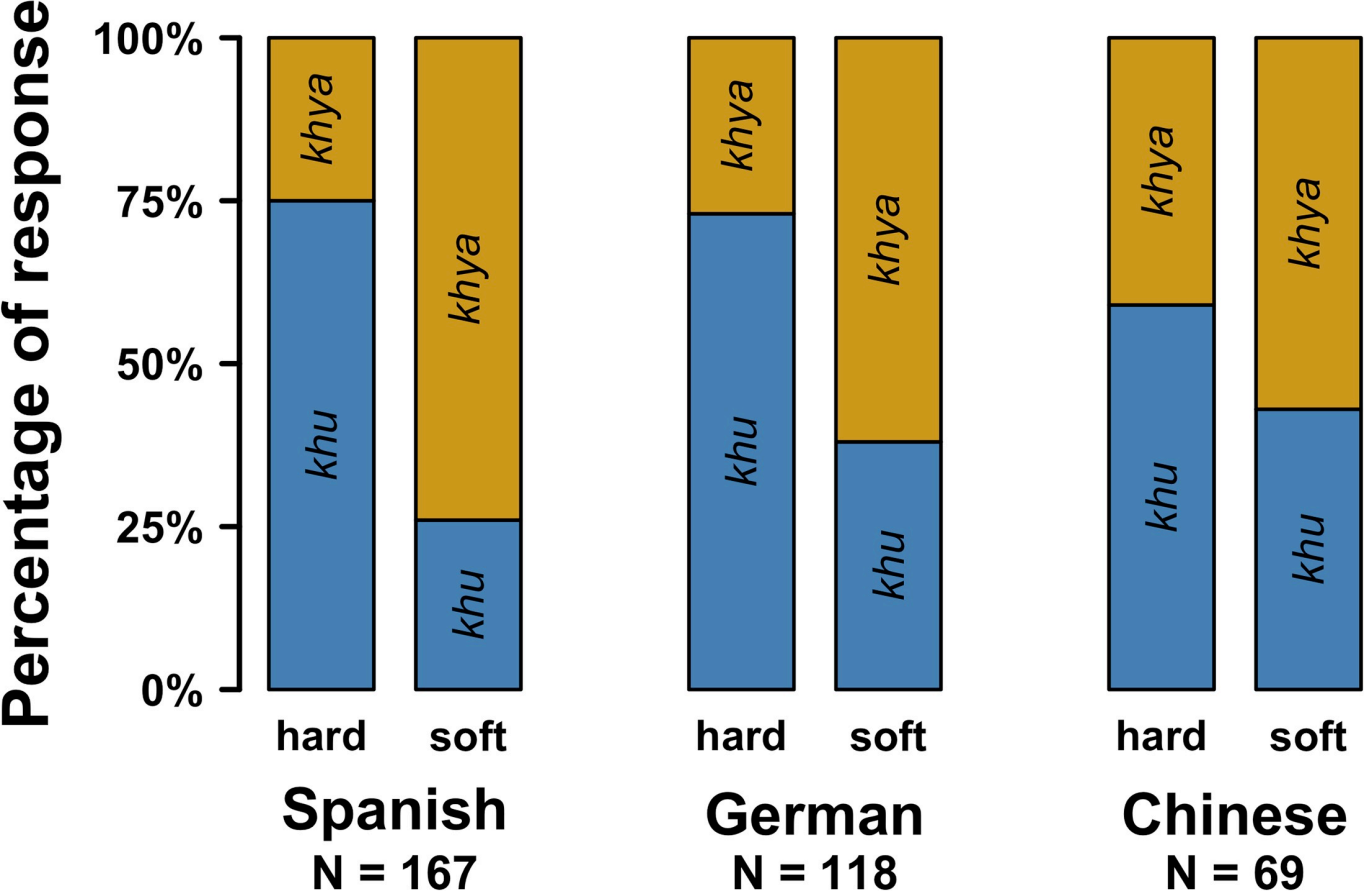
Replication of the hard/soft experiment with Spanish, German and Chinese listeners.

For the Spanish sample, 71% of all participants chose *khya* when asked to indicate the soft sound (29% chose *khu*), see Fig 2. When asked to indicate the hard sound, they chose *khya* only 24% of the time (76% chose *khu*). There was a reliable main effect of Condition (*χ*^*2*^(1) = 50.45, *p* < 0.0001), as well as a Voice Gender * Condition interaction (*χ*^*2*^(1) = 9.98, *p* = 0.002). There were no Voice Gender (*χ*^*2*^(1) = 2.69, *p* = 0.10) and Listener Gender (*χ*^*2*^(1) = 2.47, *p* = 0.12) main effects, as well as no Listener Gender * Condition interaction (*χ*^*2*^(1) = 2.81, *p* = 0.09).

For the German sample, 62% of all participants chose *khya* when asked to indicate the soft sound (38% chose *khu*). In the hard condition, only 27% of all participants chose *khya*, and 73% chose *khu*. There was a reliable main effect of Condition (*χ*^*2*^(1) = 32.09, *p* < 0.0001), as well as a Voice Gender * Condition interaction (*χ*^*2*^(1) = 12.84, *p* = 0.0003). There was a reliable Voice Gender main effect (female voices had overall slightly more *khu* responses than male voices, 55% versus 54%, *χ*^*2*^(1) = 5.9, *p* = 0.02), but no Listener Gender main effect (*χ*^*2*^(1) = 1.47, *p* = 0.22), as well as no Listener Gender * Condition interaction (*χ*^*2*^(1) = 1.06, *p* = 0.30).

For the Chinese sample, 59% of all participants chose *khya* when asked to indicate the soft sound (41% chose *khu*). In the hard condition, only 35% chose *khya*, with the remaining participants (65%) chosing *khu* instead. There was a reliable main effect of Condition (*χ*^*2*^(1) = 5.56, *p* = 0.02). There were no Voice Gender * Condition (*χ*^*2*^(1) = 1.20, *p* = 0.27) and Listener Gender * Condition (*χ*^*2*^(1) ~ 0, *p* = 0.99) interactions. There were also no Voice Gender (*χ*^*2*^(1) = 3.69, *p* = 0.05) and Listener Gender (*χ*^*2*^(1) = 0.29, *p* = 0.59) main effects.

Overall, these findings support our conclusion. Although the pattern was somewhat weaker for the Chinese sample, we were able to replicate the main finding from Experiment 1 in three additional samples, one of which is from a language unrelated to English (Chinese), and another one which is from another genus of the Indo-European language family (Spanish).

## General discussion

### Summary of findings

We have found that the Korean *khya/khu* distinction was reliably associated with softness, smoothness, gender, taste (sweet versus bitter), and pleasantness in a group of American English listeners with no knowledge of Korean. We also found partial support for an association with alcohol content. The hardness/softness experiment was replicated for Spanish, German, and Chinese listeners. The results presented in this paper support the following three claims:

- The fact that there were associations across multiple different dimensions shows that same sound pattern can be associated with multiple different perceptual and social qualities when presented in the right context. This supports the idea of latent iconicity, as well as the idea that iconicity is pluripotential.
- The contrast between the Korean interjections *khya/khu* is understood by American English, Spanish, German, and Chinese listeners, even if these listeners have little to no knowledge of the Korean language or culture. In fact, cultural geographic and cultural proximity did not appear to help the Chinese listeners, who scored the lowest. Overall, the translatability of this contrast suggests that these alcohol-related interjections used by Koreans harbor iconicity.
- The semiotic strategies used by Korean advertisers in communicating the softness of soju tap into deeply rooted cognitive associations that are shared across cultures (at least with respect to the cultures tested here).

An important aspect of our study was that we used a detailed ethnographic investigation of Korean advertising campaigns by Harkness [25] to generate predictions for our cross-linguistic listening experiments. Moreover, we used an interjection that is actually produced by Koreans in response to downing a shot of soju rather than focusing on artificial stimuli, such as the pseudowords *kiki* and *bouba*.

Across the board, we found that there were numerically stronger associations for *khu/khya* for the female voice, compared to the male voice. In some cases, there were statistically reliable interactions between the main condition manipulation and the gender of the stimulus. While this could be taken as a finding about gender (with the *khya/khu* contrast potentially being more pronounced for female voices), we have to be wary about this interpretation as we only tested one male and one female voice. This means that the factors “person” and “gender” are fully confounded. The inclusion of gender was not meant to test for gender effects as such but to show that the effect is generalizable to different kinds of voices even when we control for the voice manipulation statistically.

### Correspondences to existing iconic patterns

The finding in this study corresponds to a number of observations that have been made in the literature on iconicity in Korean and other languages. For example, Japanese participants were more likely to associate /a/ with light and smooth textures and pleasant taste characteristics [61], whereas /i/ is more likely associated with unpleasant characteristics (p. 200). The sound /a/ is what is used to transcribe *khya*, and this vocal gesture is produced with an open mouth similar to the production of this vowel. The sound /i/ is a high vowel, just as the vowel /ɯ/ in *khu*, which is a high back vowel (the unrounded version of /u/). Similarly, Fónagy [24] already noted that the high vowel /i/ may be associated with bitter tastes, compared to /a/ which is more sweet [27], see also [30]. All of these studies suggest that low, back and open vowels have overall more “positive” sensory associations (smooth, soft, sweet) than high vowels, such as /i/, or as in our study, /ɯ/ (*khu*).

It is also noteworthy that in descriptions of Korean ideophones, /ɯ/ is considered a “dark” vowel as opposed to /a/, which is considered a “bright” vowel [62–66]. Sohn [67] says that bright vowels “connote brightness, sharpness, lightness, smallness, thinness, and quickness”, whereas dark vowels “indicate darkness, heaviness, dullness, slowness, deepness, and thickness” (p. 96). In another characterization, dark vowels such as in *khu* are characterized as big, heavy, ponderous, masculine, clumsy, unwieldy, bulky, dark, and gloomy, whereas bright vowels such as in *khya* are characterized as small, affectionate, cute, feminine, fragile, flimsy, frivolous, bright, and happy [68]. It is specifically noteworthy that the vowels /ɯ/ and /a/ form a pair in the vowel harmonic system of Korean (back unrounded vowels) [66].

### Pluripotentiality: Discussion of potential mechanisms

With respect to the pluripotentiality of iconicity, the present experiments are in line with existing evidence showing that iconicity allows limited generalizability across semantic domains [69,70]. For example, Auracher [69] showed that back vowels are not only associated with the idea of large size, but also with the related idea of social dominance. Our experiments provide a direct test of this pluripotentiality across a more diverse set of semantic domains, ranging from touch-related domains (hardness, roughness), taste and pleasantness, over to alcohol content and even gender. We do not claim that *khya/khu* inherently have all of these meanings but rather that these meanings arise in a relative or contextual manner. The fact that these distinctions are not inherent to *khya/khu* can be underlined by the observation that both *khya* and *khu* are actually very “harsh” sounds, uttered with a lot of aspiration and noisy high-frequency components. Therefore, *khya* cannot communicate soft/smooth/female/low-alcohol content in an absolute sense, but rather does so in a relative or contextual way. Thus, iconicity in spoken language does not involve singular one-on-one correspondences between particular sound patterns and non-sensory meanings. Instead, iconic associations between speech sounds and meanings are multi-layered [71–74].

This picture of iconicity as something that is context-bound provides a richer description of iconic phenomena than what is sometimes assumed. Rather than being constrained to specific mappings, a resemblance between form and meaning can be *created* when the context makes particular perceptual or social dimensions sufficiently salient. In our experiments, this context was created by forcing an explicit choice between *khya* and *khu*. In Korean advertising, even though *khya* appears on its own without an explicit contrast with *khu*, the context for signaling the abstract quality of “softness” in the new soju is co-created by other semiotic resources, including the appearance of female actors talking in high-pitched voices and the light green and blue color palette. Korean speakers may also associate the innovative form *khya* with softness due to implicit knowledge that this form contrasts with *khu*, which is well-established as a vocal gesture for bitterness or disgust. Our findings therefore support Sidhu and Pexman’s [72] notion of “indirect” iconicity, which is mediated by mental associations. More broadly, the fact that social meanings such as gender are triggered indirectly through contextual usage is consistent with the notion of indexicality in anthropological linguistics [25,75].

It has to be pointed out, however, that multiple cognitive accounts could be used to explain our results. It could be that the *khya/khu* distinction taps into the same underlying core meaning which would be a highly abstract multisensory one. In this case, different shades of meaning becoming evident in particular contexts. This is akin to Rakova’s [76] “supramodal” concepts, and this idea is also implied by Harkness’s [25] analysis of the Korean ads, who speaks of “qualic transitivity.” According to him, the various semiotic channels employed by Korean advertisers (such as “voice marketing”, color palettes, female protagonists in ads) all index “the overarching abstract quality of softness” (p. 26) of the new soju. Alternatively, it could be that one of the meanings we investigated (such as, for example, the meaning of “softness”) is the primary meaning, and the other meanings are metaphoric extensions, i.e., the concept of softness is mapped onto other domains. At present, our experiment is not designed to differentiate between these cognitive accounts, but these provide fertile grounds for future research.

### Iconicity and sensory marketing

By analyzing a case of sound symbolism used in a real-world marketing strategy by major liquor brands, our results also speak to the utility of iconicity in advertising. The field of “sensory marketing” [77–79] has established the effectiveness of marketing that directly “engages the consumers’ senses” [80]. For example, Elder and Krishna [77] showed that when such food products as potato crisps and popcorn are described not only in terms of a single sense, such as taste, but also in terms of other senses such as sight, sound and touch, participants report to like the food items more. In fact, the advertising campaigns discussed by Harkness [25] can already be understood as being part of a sensory marketing strategy, as the advertisers use multiple semiotic channels to signal the same sensory quality (of abstract softness). Moreover, research on the role of metaphor in advertising has suggested that it may be advantageous to signal product qualities more indirectly, such as via metaphor [20], rather than explicitly. Given the indirect latent meanings demonstrated here, we surmise that one of the advantages of iconicity in an advertising context is that it allows “multiple, distinct, positive inferences about the advertised brand” (p. 7), just as is the case with metaphor.

### Iconicity and interjections

Our study is also important to further our understanding of interjections, which is a cover term used for expressions such as *huh*?, *ouch*, *uh-oh*, or *shh*. Dingemanse and colleagues have demonstrated the universality of *huh*?, with many different languages having the same (or a very similar-sounding) interjection [81]. In our case, it is important that American English (as well as Chinese, German and Spanish) does *not* share the interjections *khya* and *khu* tested in this study. To be sure, speakers of these languages may also produce alcohol-related sounds after downing a shot of liquor. There may also be similarities between the vowel sounds in *khu* and the English *eugh*, which is known to mark disgust [82]. However, the contrast between *khya* and *khu*—as used by Korean advertisers—is not reported for any of the languages used in this study. Nevertheless, American English listeners (as well as Chinese, German and Spanish listeners) interpret this contrast in a manner that is consistent with how the contrast is used by Korean soju advertisers, as reported in [25]. This suggests that interjections may be “motivated” (rather than arbitrary) not only for functional reasons, as suggested by Dingemanse and colleagues [81], but also via iconicity, even if this is a rather indirect form of iconicity that is mediated by semantic associations. Interestingly, researchers collecting iconicity ratings for English words [10–12] have repeatedly observed that when English speakers are asked to rate whether a word “sounds like what it means,” interjections receive very high ratings. However, these ratings are subjective and it is not clear what speakers base their iconicity judgments on. In our experiment, we provide a more direct test of the idea that an interjection (in this case, an alcohol-related interjection) can be understood across cultures even if the language in question—in this case American English—does not contain the exact same sound.

## Conclusion

To conclude, the interjective sounds that Korean speakers produce when downing a shot of soju have a diverse set of latent meanings (relating to hardness, roughness, gender, alcohol content, taste, pleasantness) that can be accessed by naïve listeners from unrelated cultures. This provides direct evidence for the idea that interjections harbor iconicity. Our results furthermore showcase the pluripotentiality of iconicity in spoken language, highlighting how sounds that are produced in the context of Korean alcohol rituals can signal a whole set of related sensory and non-sensory meanings. Finally, our results move the study of iconicity in advertising and marketing from artificial examples that involve constructed pseudowords to testing ecologically valid cases that are used in actual advertising campaigns. This approach also shows how the cross-linguistic experimental study of iconicity can take inspiration from, and subsequently contribute to detailed language- and culture-specific analyses of communicative behaviors.
